# Sulforaphane Attenuates Nonalcoholic Fatty Liver Disease by Inhibiting Hepatic Steatosis and Apoptosis

**DOI:** 10.3390/nu14010076

**Published:** 2021-12-24

**Authors:** Jinwang Li, Siyu Xie, Wendi Teng

**Affiliations:** 1 School of Food and Health, Beijing Technology and Business University, Beijing 100048, China; sdlijinwang@sina.com; 2 Key Laboratory of Functional Dairy, Ministry of Education, Department of Nutrition and Health, China Agricultural University, No. 17 Qinghua East Road, Beijing 100083, China; xiesiyu2406@163.com

**Keywords:** sulforaphane, hepatic steatosis, apoptosis, lipid, nonalcoholic fatty liver disease

## Abstract

Nonalcoholic fatty liver disease (NAFLD) is characterized by lipotoxicity and ectopic lipid deposition within hepatocytes. Sulforaphane (SFA), an active compound used for inhibiting tumors, was found to have the potency to improve lipid metabolism. However, its molecular mechanisms on ameliorating NAFLD are still incompletely understood. This research evaluated if SFA could inhibit hepatic steatosis and apoptosis. The effects of SFA on cell viability, lipid accumulation, triglyceride (TG) contents, apoptosis, ceramide contents, and reactive oxygen species (ROS) levels were analyzed in palmitic acid (PA)-treated HepG2 cells and high-fat diet (HFD)-fed mice. The related molecular mechanisms were further explored in hepatocytes. The results showed SFA alleviated lipid accumulation and regulated AMPK/SREBP1c/FAS signaling pathway in PA-stressed HepG2 cells. In addition, SFA alleviated PA-mediated apoptosis, downregulated the expressions of cleaved caspase 3, as well as reduced ceramide contents and ROS levels. Moreover, SFA treatment reduced HFD-induced body weight gain, alleviated insulin resistance, decreased serum TG, total cholesterol (TC), and alanine aminotransferase (ALT) levels, and prevented lipid deposition and apoptosis in the liver. This study showed SFA suppressed lipid deposition and apoptosis both in vitro and in vivo, indicating that SFA may be a potential candidate for preventing and treating NAFLD.

## 1. Introduction

Nonalcoholic fatty liver disease (NAFLD) is characterized by the excessive lipid deposition in hepatic cells (more than 5% fat contents in the liver) and describes a spectrum of complicated pathological processes, such as nonalcoholic simple fatty liver, nonalcoholic steatohepatitis, liver fibrosis, and even predisposes one to liver-related complications, such as cirrhosis and hepatocellular carcinoma [1]. NAFLD is the most frequent cause of liver diseases around the world, with an increasing population from 17% to 46% in Western countries [2,3]. The incidence of NAFLD is rapidly increasing due to its strong association with obesity, dyslipidemia, insulin resistance, type 2 diabetes mellitus, as well as cardiovascular disease [4].

Though the mechanisms are still unclear, a “two hits” model has been proposed for the pathogenesis of NAFLD. Hepatic excess fat accumulation is the “first hit”, which is a progression of triglyceride accumulation induced by the imbalance between the import and synthesis of hepatic lipids on the one hand, and β-oxidation and outflux on the other [5]. The “second hit” is caused by oxidative stress and inflammation, resulting in further damage and apoptosis in the liver [6]. Many clinical and animal research studies have shown the pivotal roles of lipid deposition and apoptosis in the progression of NAFLD [7,8]. Despite many efforts, there is still no approved drug for treating NAFLD. Currently, developed pharmacotherapy showed limited beneficial effects and some side effects. For example, pioglitazone, an insulin sensitizer, diverts the non-esterified free fatty acid (FFA) load toward adipocytes instead of the liver. Nevertheless, it would lead to weight gain, bone fractures in women, congestive heart failure, and an increased risk of bladder cancer [9,10]. Therefore, the discovery of functional foods or food-derived active ingredients with the least side effects could offer an effective strategy to ameliorate and treat NAFLD [11].

Sulforaphane (SFA), an isothiocyanate found in cruciferous vegetables, was firstly used for inhibiting tumors [12,13]. Recently, emerging scientific studies showed SFA could reduce hepatic glucose production [14] and improve lipid profile in HepG2 cells and mice [15]. Though nuclear factor erythroid-related factor 2 (Nrf2) was proved to be a target of SFA for improving hepatic architectural integrity and functioning against pathological states [16], precise mechanisms contributing to its functions in NAFLD must be further explored. The purpose of this research was to assess the effects of SFA on ameliorating hepatic steatosis and apoptosis in vitro and in vivo and develop a further understanding of SFA against NAFLD progression.

## 2. Materials and Methods

### 2.1. Materials and Chemicals

SFA (purity > 99%) was obtained from LKT Laboratories (St. Paul, MN, USA). Human recombinant insulin and palmitic acid (PA) were purchased from Sigma-Aldrich, USA. Annexin V-FITC apoptosis detection kit was from Life Technologies (Carlsbad, CA, USA). BCA protein concentration determination kit, total cholesterol (TC) assay kit, triglycerides (TG) assay kit, aspartate aminotransferase (AST) assay kit, and alanine aminotransferase (ALT) assay kit were from Nanjing Jiancheng Bioengineering Institute, China. All chemicals used in the research were of analytical grade.

### 2.2. Cell Culture 

Human hepatic carcinoma, HepG2 cells, were obtained from Beijing Union Medical Cell Resource Center (Basic Medical Cell Center, Institute of Basic Medical Sciences, Chinese Academy of Medical Sciences), and were cultured in minimum essential medium (MEM) supplemented with 10% fetal bovine serum, 1% nonessential amino acids, streptomycin (100 µg/mL) and penicillin (100 U/mL) (Invitrogen, Carlsbad, CA, USA) C in a 37 °C incubator with 5% CO_2_ and 95% humidity. 

### 2.3. Cell Viability Assessment 

The HepG2 cells viability was measured by cell counting kit-8 (CCK-8) colorimetric assay (Beyotime, Haimen, Jiangsu, China), according to the manufacturer’s instructions. Briefly, cells were subcultured in 96-well plates at 1 × 10^4^ per well. After treatments, CCK8 reagent was added to each well, followed by incubating for another 1 h in a 37 °C incubator with 5% CO_2_. Then, the absorbance was measured by a microplate reader at 450 nm (Bio-Rad, Hercules, CA, USA), and cell viability was expressed as percentage values, as compared with the control group.

### 2.4. Cell Apoptosis Assay

Cells were seeded at a density of 3 × 10^5^ cells per well into 6-well plates. After treatments, the cells were washed twice with cold phosphate-buffered saline (PBS, pH 7.4) and then resuspended in 1× binding buffer at a concentration of 5 × 10^5^ cells/mL. Then, 5 µL annexin V and 10 µL PI were separately added into each tube. After 15 min incubation at room temperature and in the dark, 400 µL of 1× binding buffer was added to each tube. Cell fluorescence was detected by FACS CytoFLEX flow cytometer (Beckman Coulter, S. Kraemer Boulevard Brea, CA, USA). Data were analyzed using CytExpert 2.0.0 software (Beckman Coulter, S. Kraemer Boulevard Brea, CA, USA).

### 2.5. Determination of Intracellular ROS 

The intracellular ROS was analyzed with fluorescent probe 2, 7-dichlorodihydrofluorescein diacetate (DCFH-DA), as described previously [16]. After treatments, cells were washed once with PBS. Then, 10 µM DCFH-DA was added to the cells. After 30 min incubation at 37 °C, the cells were washed twice with PBS to remove excessive DCFH-DA. Finally, cells were trypsinized and resuspended in PBS. ROS levels were determined by FACS CytoFLEX flow cytometer (Beckman Coulter, S. Kraemer Boulevard Brea, CA, USA) at 488 nm excitation wavelength and 525 nm emission wavelength. Data were analyzed by CytExpert 2.0.0 software (Beckman Coulter, S. Kraemer Boulevard Brea, CA, USA).

### 2.6. Measurement of Intracellular Lipid Content

HepG2 cells were fixed in 4% paraformaldehyde for 15 min, washed with PBS, and incubated with Oil Red O working solution for 15 min at room temperature. Then, lipid droplets were observed and photographed with a microscope (Carl Zeiss, Jena, Germany). To measure intracellular lipid content by BODIPY lipid probes (Invitrogen, Carlsbad, CA, USA), HepG2 cells were incubated with 10 μg/mL of BODIPY for 10 min at 37 °C and then stained with DAPI for 3 min. After washing three times with PBS, cells were immediately visualized using a Zeiss LSM-700 confocal microscope (Carl Zeiss, Jena, Germany).

### 2.7. Intracellular TG Assay

TG contents were determined enzymatically using a commercially available kit according to the manufacturer’s instructions. Intracellular TG content was normalized according to the protein concentration and reported as mmol of TG/g of protein.

### 2.8. Animals and Experimental Design 

For this study, 6-week-old male C57BL/6J mice were obtained from Beijing Vital River Laboratory Animal Technology Co (Beijing, China). Mice were first housed in a controlled environment (temperature, 23 ± 1 °C; humidity, 60%) under a 12 h light/dark cycle. One week later, mice were randomly allocated to three groups as follows: control group (*n* =  8) was fed with normal chow (NC; 12450b, Research Diets, NJ, USA, 10% kcal fat content) for 17 weeks; HFD group (*n* =  8) was fed with a high-fat diet (HFD; D12492, Research Diets, NJ, USA, 60% kcal fat content) for 17 weeks; HFD +  SFA group (*n* =  8) received HFD for 17 weeks and were intraperitoneally injected with 10 mg/kg/day SFA from week 14 to week 17 additionally (three times per week). After 17 weeks, mice were fasted overnight and anesthetized. Blood samples were taken from the vena cava. Livers were immediately excised, weighed, and stored at −80 °C for further molecular experiments. The whole experiment was performed in accordance with the Guide for the Care and Use of Laboratory Animals and the research protocol was approved by the Experimental Animal Ethics Committee in China Agricultural University (Permission Number: Aw71211202-4-2).

### 2.9. Body Fat Distribution and Content 

Fat distribution and contents of mice (*n* = 6) were evaluated under anesthesia using 4% chloral hydrate (4 mL/kg) by MesoMR23-060H-I imaging instrument (Shanghai Niumag Corporation, Shanghai, China). The parameters were set as: K space = 192 × 256 mm, time of echo = 13.5 ms, time of waiting = 300 ms, magnet = 0.55 T, section thickness = 3.5 mm, field of view read = 100 mm, field of view phase = 100 mm and number of scans = 8. The body contents can be obtained from the image analysis based on the measurement.

### 2.10. Histopathology 

For analysis of hematoxylin and eosin-stained liver sections, liver tissues were fixed in 4% paraformaldehyde for 72 h and then embedded in paraffin. To visualize lipid droplets in the liver, the frozen liver sections were collected and were subjected to Oil red O staining. The images were captured and analyzed using a Zeiss LSM-700 confocal microscope (Carl Zeiss, Jena, Germany).

### 2.11. Biochemical Analyses of Liver Tissues

Liver tissues were homogenized in cold 50 mM Tris-HCl buffer (pH 7.4) containing 1 mM EDTA and centrifuged at 12,000× *g* for 30 min at 4 °C. The supernatants were collected to analyze TG, TC, ALT, and AST contents in the liver using biochemical assay kits according to the manufacturer’s instructions.

### 2.12. Glucose Tolerance Test and Insulin Tolerance Test

Mice were fasted for 12 h to analyze the glucose tolerance test after an intraperitoneal injection of 2 g/kg glucose. For the insulin tolerance test, mice were intraperitoneally injected with 1 IU/kg insulin (Eli Lilly and Co., Humalog Insulin) after 6 h fasting. Blood samples from the tail veil were measured with a glucometer (TheraSense Freestyle, CA, USA) at the indicated times (0, 30, 60, 90, 120, and 180 min) after injection.

### 2.13. Ceramide Content Analysis 

After treatment, cells suspension and liver homogenate were collected separately. After protein determination, 50 µL internal standard solutions were added to the samples, and 2 mL extraction mixture (isopropanol:water:ethyl acetate = 30:10:60; *v:v:v*) was used for lipids extraction. The samples were vortexed, sonicated 30 s for 3 times, and centrifuged for 10 min at 4000× *g*. The supernatants were transferred and reextracted as described above. The extracted samples were dried by a gentle nitrogen stream. Then, quantification of ceramides was performed on an Agilent high-performance liquid chromatography (HPLC) system, coupled with a quadrupole-time of flight mass spectrometer (6545 Q-TOF), as described previously [17,18], based on the C17-ceramide internal standards from Avanti Polar Lipids (Alabaster, AL, USA), as well as C14-ceramide, C18-ceramide, C20-ceramide, C22-ceramide, C24-ceramide, C24:1-ceramide, and C26:1-ceramide standards from ZZStandard (Shanghai, China). Ceramide level is expressed as µmol/protein (mg/mL).

### 2.14. TUNEL Assay

An assay for terminal deoxynucleotidyl transferase-mediated dUTP nick end labeling (TUNEL, Roche Diagnostics, Germany) was conducted to detect hepatocyte apoptosis [19]. The hepatic tissue slices of different groups were conventional dewaxing and rehydration, and apoptosis was measured by TUNEL assay. Under an ordinary optical microscope (Carl Zeiss, Jena, Germany), the positive apoptotic cells showed reddish brown.

### 2.15. Western Blotting 

Western blots were performed as described previously [20] for AMP-activated protein kinase (AMPK; Cell Signaling Technology, Danvers, MA, USA), p-AMPK (Cell Signaling Technology), cleaved caspase 3 (Cell Signaling Technology), sterol regulatory element-binding proteins 1c (SREBP1c; Abcam, Cambridge, UK), β-actin (Abcam), and fatty acid synthase (FAS; Santa Cruz Biotechnology, CA, USA).

### 2.16. Statistical Analysis 

All assays were performed at least in triplicate, and data were presented as means ± standard deviations (SDs). Significant differences (*p* < 0.05) between means were assessed using one-way ANOVA, followed by Duncan’s multiple-comparison test by SPSS 20.0 software (IBM Inc., Chicago, IL, USA).

## 3. Results

### 3.1. SFA Alleviated PA-Mediated Cytotoxicity in HepG2 Cells 

**Figure 1:**
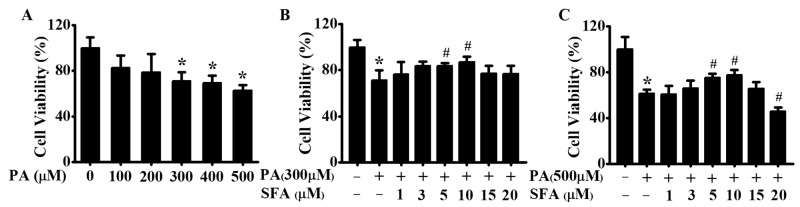
Effects of PA and SFA on cell viability in HepG2 cells: (**A**) cells treated with different concentrations of PA for 24 h. Cells treated with different concentrations of SFA in the presence of 300 µM of PA (**B**) or 500 µM of PA (**C**) for 24 h. Cell viability presented as the percentage of viable cells relative to that in control group. All values are expressed as means ± SD from three independent determinations, * *p* < 0.05 vs. control cells; # *p* < 0.05 vs. only PA-stressed HepG2 cells. PA, palmitic acid; SFA, sulforaphane.

To investigate the potential of PA to induce cell death, a CCK-8 assay was performed. HepG2 cells were first treated with rising doses of PA (0, 100, 200, 300, 400, or 500 µM) for 24 h, and cell viability was evaluated. As shown in Figure 1A, cell viability was significantly decreased after treatment with 300, 400, or 500 µM of PA. To further explore the role of SFA on PA-induced cell death, its effect on cell viability was next measured. As shown in Figure 1B, compared with the control group, 300 µM of PA remarkably decreased the survival of HepG2 cells. However, the decrease was ameliorated by SFA (1, 3, 5, or 10 µM) in a dose-dependent manner, especially by 5 or 10 µM of SFA. Similarly, 5 or 10 µM of SFA remarkably ameliorated 500 µM of PA-induced HepG2 cells death (Figure 1C). These results suggested that SFA alleviated PA-mediated cytotoxicity in HepG2 cells, and we thus chose 5 and 10 µM of SFA in the following experiments.

### 3.2. SFA Attenuated PA-Induced Lipid Accumulation in HepG2 Cells

**Figure 2:**
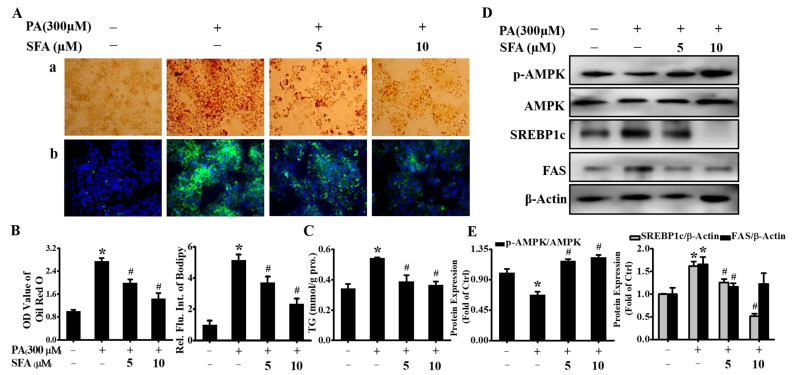
Effects of SFA on lipid accumulation and related protein expression in PA-stressed HepG2 cells: (**A**) evaluation of lipid accumulation by Oil red O staining (**a**) and BODIPY staining (**b**) after different concentrations of SFA treatments in the presence of 300 µM PA for 24 h; (**B**) quantitative analysis of lipid accumulation based on the imaging in (**A**) by colorimetric assays; (**C**) the measurement of TG contents (mmol/g protein) with different treatments. Western blot analysis showed the expressions of phosphorylated AMPK, total AMPK, SREBP1cm and FAS (**D**), and gray analysis of each band (**E**). All values are expressed as means ± SD from three independent determinations, * *p* < 0.05 vs. control cells: # *p* < 0.05 vs. only PA-stressed HepG2 cells.

The effect of SFA on PA-induced lipid accumulation was measured by Oil Red O and BODIPY staining, and the extents of staining were spectrophotometrically quantitated. As shown in Figure 2A, lipid droplets were observed in PA-treated HepG2 cells, whereas SFA significantly reduced PA-induced lipid deposition. Interestingly, the extent of reduction in staining significantly increased as doses of SFA rose, and the ratios of cells’ staining were 1, 2.74, 1.99, and 1.43, respectively, with Oil Red O staining; 1, 5.15, 3.72, and 2.35, respectively, with BODIPY staining (Figure 2B). Our data also found PA significantly increased intracellular TG contents (Figure 2C). However, SFA remarkably decreased the TG levels, showing a similar pattern with the results in Oil Red O and BODIPY staining. These data suggested that PA-induced lipid accumulation in HepG2 cells was dose-dependently decreased by SFA treatment.

In order to further understand the possible mechanisms behind the lipid-reducing effect of SFA, the phosphorylated AMPK in HepG2 cells was measured. The level of p-AMPK was reduced to 0.67 after incubation with 300 µM PA for 24 h but returned to 1.17 and 1.22 under co-incubation with 5 or 10 µM of SFA, respectively (Figure 2D,E). The transcription factors SREBP1c and FAS have been identified as important targets of AMPK. Upon AMPK activation, SREBP1c and FAS are downregulated, ultimately leading to lipid synthesis inhibition [21,22]. Our results showed that SREBP1c and FAS were significantly increased when HepG2 cells were incubated with 300 µM PA, whereas SFA largely prevented the increase (Figure 2D,E). Taken together, these results implied that SFA led to the AMPK activation and downregulation of SREBP1c and FAS, thus preventing hepatic lipid deposition.

### 3.3. SFA Ameliorated PA-Stressed Apoptosis in HepG2 Cells

**Figure 3:**
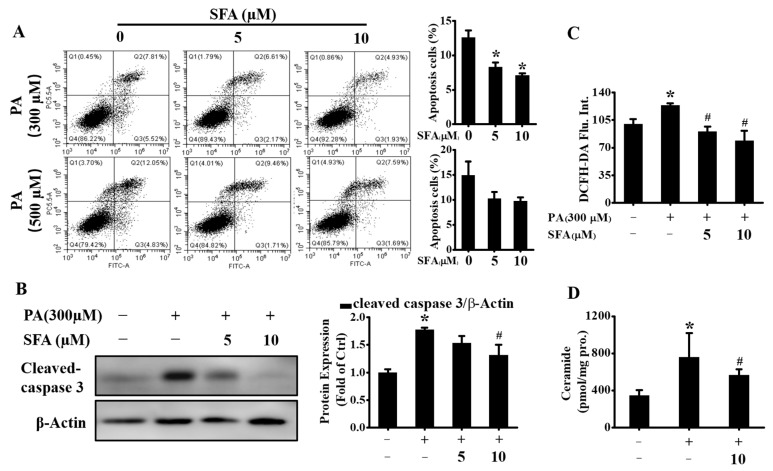
Effects of SFA on alleviating PA-induced apoptosis in HepG2 cells: (**A**) apoptosis detected by annexin V (FITC)/PI staining and the percentage of apoptotic cells was calculated; (**B**) Western blot analysis showed the expressions of cleaved caspase 3 and gray analysis of each band; (**C**) ROS levels evaluated by DCFH-DA staining and expressed as mean fluorescence intensity relative to that in control group; (**D**) total ceramide contents detected by LC/MS/MS. All values are expressed as means ± SD from three independent determinations. * *p* < 0.05 vs. control cells; # *p* < 0.05 vs. PA-treated cells.

Apoptosis is one of the main types of cell death and plays a fundamental role in the development of multicellular organisms [23]. Saturated FFA-induced hepatocyte apoptosis is a feature of NAFLD [24]. Here, whether SFA alleviated PA-mediated cytotoxicity by apoptosis inhibition was determined. The results by flow cytometric analysis of annexin V-FITC/PI staining confirmed that the exposure to 300 or 500 µM PA induced obvious apoptosis in HepG2 cells (Figure 3A). However, the apoptosis cells were markedly decreased when co-incubation with 5 or 10 µM SFA. To further verify the antiapoptosis effect, the protein expressions of cleaved caspase 3 were assayed. Consistent with the annexin V-FITC/PI assay results, cleaved caspase 3 was upregulated after PA treatment, whereas the co-incubation with SFA suppressed the cleavage of caspase 3 (Figure 3B), suggesting that SFA ameliorated PA-induced apoptosis in HepG2 cells.

Excess oxidative stress is also a well-characterized mechanism of PA-induced hepatic lipotoxicity and the increased intracellular ROS results in hepatocyte apoptosis [25]. Thus, the effect of SFA on PA-induced oxidative stress by flow cytometric analysis of DCFH-DA staining was determined. As shown in Figure 3C, PA-induced ROS increase was dose-dependently reduced by SFA. In addition, the PA-stressed increase in ceramide contents was lowered by SFA (Figure 3D), which as a second messenger can trigger apoptosis in steatohepatitis [26]. Together, these data further revealed that SFA attenuated PA-induced apoptosis in HepG2 cells.

### 3.4. SFA Alleviated HFD-Induced Body Weight Gain and Insulin Resistance in C57BL/6J Mice

**Figure 4:**
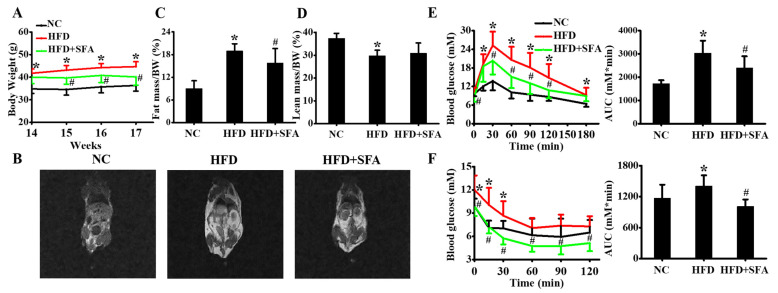
Inhibition effects of SFA on weight gain, fat distribution and insulin resistance in HFD-fed C57BL/6J mice: (**A**) Body weight of mice in three groups from week 14 to week 17 (*n* = 8/group); (**B**) MRI images showed fat distribution in mice at week 17 (*n* = 6/group). The percentage of fat mass (**C**) and lean mass (**D**) were calculated (*n* = 6/group); (**E**) glucose tolerance test and the area under the curve were analyzed at week 17 (*n* = 8/group); (**F**) insulin tolerance test and the area under the curve were analyzed at week 17 (*n* = 8/group). Values All values are expressed as means ± SD, * *p* < 0.05 vs. NC group; # *p* < 0.05 vs. HFD group. NC, normal chow; HFD, high-fat diet; AUC, area under the curve.

In order to explore the effects of SFA on NAFLD in vivo, SFA administration experiments in C57BL/6J mice were conducted. The effects of SFA on body weight and fat distribution were first assessed. After 14 weeks’ treatment with NC or HFD, HFD-fed mice markedly gained body weight, compared with the NC group. Surprisingly, after another 3 weeks of 10 mg/kg SFA intervention, body weight gain was significantly decreased in mice (Figure 4A). This reduction was probably attributed to the decreased fat mass and moderately increased lean mass (Figure 4B–D). Meanwhile, SFA significantly suppressed serum TG, TC, and ALT levels in HFD-fed mice (Table 1). The effects of SFA on blood glucose were also examined, and the data showed 10 mg/kg SFA remarkably decreased the blood glucose levels, in both glucose tolerance (Figure 4E) and insulin tolerance tests (Figure 4F). These data suggested that HFD led to obesity and insulin resistance, but SFA successfully reversed the changes.

### 3.5. SFA Reduced HFD-Induced Hepatic Steatosis and Apoptosis in C57BL/6J Mice

**Figure 5:**
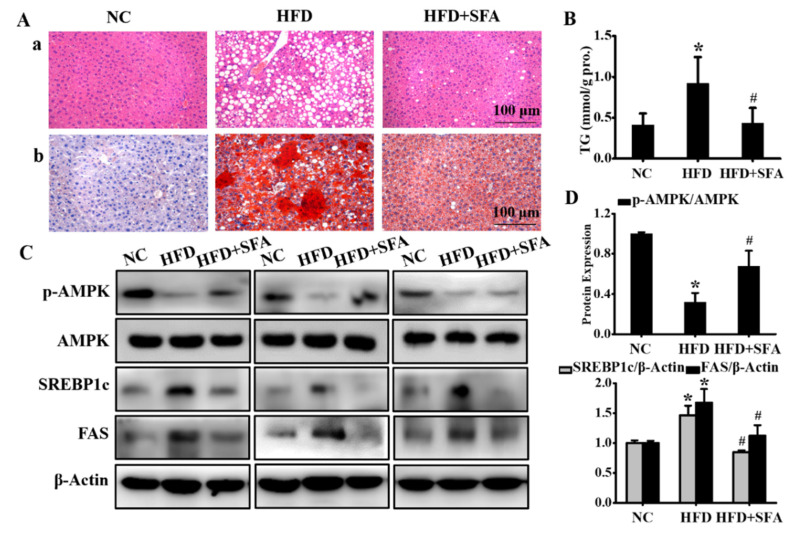
Evaluation of hepatic steatosis after SFA treatment to HFD-fed C57BL/6J mice: (**A**) histological images of liver sections by H&E (**a**) and Oil red O (**b**) staining. (*n* = 5/group); (**B**) determination of TG levels in liver. (*n* = 5/group); (**C**) Western blot analysis showed the expressions of phosphorylated AMPK, total AMPK, SREBP1c, and FAS in liver; (**D**) gray analysis of each band (*n* = 3/group). All values are expressed as means ± SD, * *p* < 0.05 vs. NC group; # *p* < 0.05 vs. HFD group.

To further evaluate the effects of SFA on HFD-induced hepatic lipid deposition, H&E and Oil Red O staining of liver sections were analyzed. The results showed hepatocellular ballooning and hepatocyte fatty degeneration in the HFD group, whereas the lipid droplets were significantly reduced after SFA administration (Figure 5A). Moreover, SFA significantly decreased the hepatic TG levels in HFD-fed mice (Figure 5B). Signaling pathways involved in lipid accumulation were also examined. Consistent with the in vitro results, the in vivo results showed that SFA reversed the protein levels of SREBP1c, FAS, and phosphorylated AMPK in HFD-fed mice (Figure 5C,D). 

**Figure 6:**
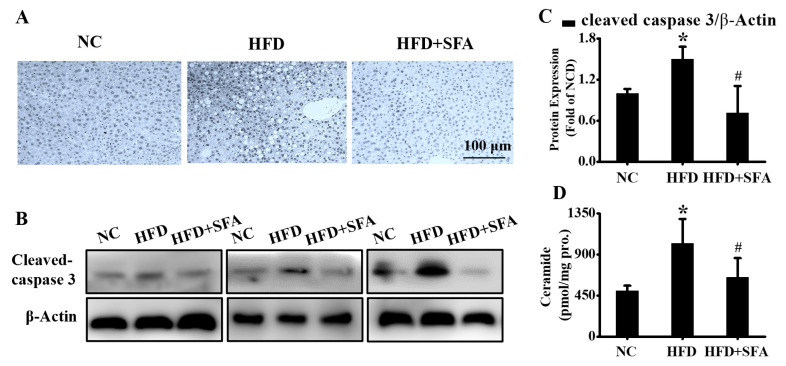
Evaluation of hepatic apoptosis and ceramide contents after SFA treatment to HFD-fed C57BL/6J mice: (**A**) the hepatic apoptosis in different groups examined by TUNEL assay. (*n* = 5/group); (**B**) Western blot analysis showed the levels of cleaved caspase 3 and (**C**) gray analysis of each band in liver (*n* = 3/group); (**D**) total ceramide contents in liver detected by LC/MS/MS (*n* = 4–5/group). All values are expressed as means ± SD, * *p* < 0.05 vs. NC group; # *p* < 0.05 vs. HFD group.

Next, the apoptosis in liver tissues was measured by a TUNEL assay. As shown in Figure 6A, the hepatic apoptosis in the HFD group was significantly increased compared with that in the NC group (*p* < 0.05), whereas SFA remarkably recovered these changes. In addition, the increased cleaved caspase 3 was downregulated by SFA (Figure 6B,C). Furthermore, HFD significantly elevated hepatic ceramides (Figure 6D). However, treatment with SFA largely lowered ceramide contents by the end of the administration period. Altogether, these data strongly indicated that SFA attenuated HFD-induced hepatic steatosis and apoptosis in C57BL/6J mice.

## 4. Discussion

This study, for the first time, found that SFA significantly alleviated apoptosis in the development of NAFLD. In addition, a potential new health-related mechanism for SFA was suggested. SFA alleviated lipid accumulation by regulating the AMPK/SREBP1c/FAS signaling pathway. Moreover, antiapoptotic activity, accompanied by a reduction in ceramide production and ROS levels, was evaluated. These results suggest that SFA, as a functional ingredient, could have a substantial impact preventing NAFLD progression.

NAFLD patients are shown to have increased lipolysis and carried FFAs to the liver. Excessive FFAs accumulation is the principal contributor to lipotoxicity because of the potential cellular toxins and leads to excessive lipid steatosis [27]. Therefore, in the present study, saturated long-chain fatty acid PA was used to successfully induce lipotoxicity and lipid accumulation in HepG2 cells. As a natural, active compound originally used for anticancer therapy [17,18], SFA has recently been suggested to alternatively treat metabolic diseases [14,15,28]. In cell studies, 2.5–10 µM SFA promoted lipolysis via activating hormone-sensitive lipase in adipocytes [29]. In hepatocytes, treatment with 0.5–10 µM of SFA for 24 h decreased glucose production [16]. This study showed that 1, 3, 5, or 10 µM of SFA alleviated PA-induced cell death, but 15 or 20 µM of SFA even inhibited HepG2 cells growth when co-incubation with 500 µM of PA. These results revealed that relatively low doses of SFA (<10 µM) could induce additional actions relevant to metabolic diseases, whereas higher doses (>10 µM) of SFA showed effective antitumor treatments, which were proved by a considerable number of preclinical studies [30,31]. 

NAFLD is mainly characterized by fat deposition in hepatocytes, visible at light microscopy as small droplets inside the cytoplasm. Thus, the therapy based on reducing lipid accumulation is ideal for treating NAFLD [32]. It is reported that SFA activates lipolysis by transcriptionally upregulating adipose triglyceride lipase and hormone-sensitive lipase in HHL-5 cells [33]. In the current study, we observed that SFA inhibited lipid deposition and reduced TG contents in PA-treated HepG2 cells. In addition, SFA treatment prevented lipid deposition in the liver, decreased serums TG, TC, and ALT levels, and also reduced HFD-induced weight gain and fat distribution in mice. The molecular mechanism of SFA-attenuating lipid accumulation was further explored. AMPK, as an energy sensor, contributes to keeping cellular energy homeostasis [34]. Activated AMPK abolishes the lipid synthesis process and reduces TG production in the liver [35]. Studies have indicated that AMPK suppresses lipogenesis-associated genes, such as SREBP1c and FAS. SREBP1c, as a critical factor involved in hepatic lipid synthesis, also regulates key genes involved in hepatic lipogenesis, including FAS [36]. The results showed SFA reversed the decline of phosphorylated AMPK, followed by the downregulation of SREBP1c and FAS, thus preventing hepatic TG synthesis both in the cell and mice model of NAFLD. A similar effect was also observed after administration with troglitazone in the hearts of obese Zucker rats and ob/ob mice [37]. Collectively, this research concludes that SFA could reduce hepatic de novo lipogenesis and inhibit excessive lipid accumulation by regulating the AMPK/SREBP1c/FAS signaling pathway.

Apoptosis is defined as programmed cell death and is accompanied by many specific morphological features, such as cell shrinkage, nuclear fragmentation, and chromatin condensation, which is another important mechanism in the progression of NAFLD. Hepatocyte apoptosis is often observed in NAFLD studies [38,39]. When saturated FFAs exceed the hepatic storage limit, they activate apoptosis signaling [40]. Caspases are a family of cysteine proteases, and the caspase pathway has been implicated as a primary mechanism of hepatocyte apoptosis. Several studies have suggested that caspase inhibitors or specific depletion of caspase can effectively suppress apoptosis both in vitro and in animal models, suggesting that targeting specific caspase is a viable approach [41,42]. Caspase 3 is an indispensable caspase for chromatin condensation and DNA fragmentation, which are the final steps of apoptosis [43]. In the present study, the number of apoptosis cells and cleaved caspase 3 expressions were remarkably increased in PA-treated HepG2 cells, confirming the pathological mechanism underlying the apoptosis. Interestingly, the data showed that SFA repressed apoptosis and decreased the cleaved caspase 3 in vitro and in vivo, suggesting that SFA could protect from the development of NAFLD. Ceramides are a class of sphingolipids that exist in membranes and function as intracellular signaling molecules [44]. Mounting bodies of evidence indicate that ceramides also act as a second messenger of the FFA-induced apoptosis effect. PA, a precursor in de novo ceramide synthesis, induces ceramide production. Ceramides subsequently disturb electron transport in the mitochondrial respiratory chain complexes I and III [45], which results in ROS generation, followed by cytochrome c release into the cytosol and caspase-induced apoptosis [46]. Inhibition of ceramide synthesis by fumonisin B1, a ceramide synthase inhibitor, attenuated PA-induced apoptosis in rodent and human β cells [47,48]. In the current study, we found that PA increased the ceramide production and ROS levels in a NAFLD state. However, the increases in ceramide and ROS contents were reversed by SFA treatment. These data together provide support for the role of SFA in alleviating hepatocyte apoptosis. This study is the first to provide evidence that SFA significantly alleviated the development of NAFLD by preventing apoptosis. In addition, the deeper molecular mechanism of the antiapoptosis effect by SFA still needs to be further investigated.

In conclusion, the data reveal that SFA suppresses PA-induced lipid accumulation and apoptosis in HepG2 cells, as well as HFD-induced liver injury and steatosis in C57BL/6J mice. This work contributes a new understanding with regard to the molecular mechanisms underlying the inhibitory effect of SFA on hepatic lipid deposition and apoptosis. This study suggests that SFA may be a potential candidate for the amelioration and prevention of NAFLD.

## Figures and Tables

**Table 1:** Metabolic parameters of serum in C57Bl/6J mice.

	NC	HFD	HFD + SFA
TG (mmol/L)	0.81 ± 0.07	1.73 ± 0.27 *	1.43 ± 0.31 ^#^
TC (mmol/L)	2.3 ± 0.17	4.25 ± 1.03 *	3.1 ± 0.6 ^#^
ALT (U/L)	36.6 ± 3.74	99.81 ± 37.09 *	48.31 ± 20.53 ^#^
AST (U/L)	223.07 ± 20.82	322 ± 117.99	249.72 ± 59.62

TG, TC, ALT, and AST levels measured in fasting plasma. Measurements are presented as means ± SEM (*n* = 4–6/group). All values are expressed as means ± SD, * *p* < 0.05 vs. NC group; ^#^ *p* < 0.05 vs. HFD group.

## Abbreviations

ALT: alanine aminotransferase; AMPK: AMP-activated protein kinase; AST: aspartate aminotransferase; CCK-8: cell counting kit-8; DCFH-DA: dichlorodihydrofluorescein diacetate; FAS: fatty acid synthase; FFA: free fatty acid; HFD: high-fat diet; HPLC: high-performance liquid chromatography; NAFLD: nonalcoholic fatty liver disease; NC: normal chow; PA: palmitic acid; PBS: phosphate-buffered saline; ROS: reactive oxygen species; SFA: sulforaphane; SREBP1c: sterol regulatory element-binding proteins 1c; TC: total cholesterol; TG: triglyceride; TUNEL: terminal deoxynucleotidyl transferase-mediated dUTP nick end labeling.
